# Lipomas as an Extremely Rare Cause for Brachial Plexus Compression: A Case Series and Systematic Review

**DOI:** 10.1055/s-0041-1726087

**Published:** 2021-04-13

**Authors:** Oliver Gembruch, Yahya Ahmadipour, Mehdi Chihi, Thiemo F. Dinger, Laurèl Rauschenbach, Daniela Pierscianek, Ramazan Jabbarli, Ulrich Sure, Karsten H. Wrede, Anne-Kathrin Uerschels

**Affiliations:** 1 Department of Neurosurgery, University Hospital Essen, University of Duisburg-Essen, Essen, Germany

**Keywords:** brachial plexus, lipoma, surgery, intraoperative, monitoring

## Abstract

**Introduction** Brachial plexus lipomas are extremely rare benign tumors that may cause slow progression of neurological deficits leading to thoracic outlet syndrome. Up to now, surgery remains challenging. The aim of this study is to present our surgical treatment regime and long-term neurological outcome in three cases of giant brachial plexus lipomas and to show results of systematic review.

**Patients and Methods** Retrospective analysis of our database “peripheral nerve lesion” to identify patients suffering from brachial plexus lipomas between January 1, 2012, and December 31, 2019. Systematic review was performed for literature published until March 31, 2020, analyzing PubMed, Google Scholar, Scopus, and the Cochrane Collaboration Library independently by two authors.

**Results** Over the past years, three patients suffering from giant brachial plexus lipomas attended to our neurosurgical department. All patients underwent preoperative magnetic resonance imaging (MRI), ultrasound examinations, and electrophysiological testing. Tumors were removed microsurgically via anterior/posterior, supraclavicular/infraclavicular, and combined approaches. The patients were accessed postoperatively by MRI and clinical follow-up. Systematic review of the literature revealed 22 cases, which were analyzed in regard to demographics, surgical treatment, and neurological outcome.

**Conclusion** Brachial plexus lipomas are an extremely rare cause for brachial plexus compression. Total microsurgical removal with intraoperative electrophysiological monitoring is the treatment of choice with excellent long-term MRI and clinical outcome.

## Introduction

Brachial plexus tumors are relatively rare, and treatment is often challenging due to the complex anatomical location with adjacent nervous and vascular structures. In the majority of the cases, lipomas are benign asymptomatic soft tissue tumors showing only slow progression. Nevertheless, in very rare cases, deep-seated lipomas can grow to gigantic size measuring more than 10 cm of length 1 before causing symptoms such as nerve compression-associated motor deficits, sensory alterations, pain, or visceral symptoms. 2 The brachial plexus compression can lead thoracic outlet syndrome (TOS)-like symptoms in some cases. A diverse group of disorders related to extrinsic compression of neurovascular structures between the first rib and the clavicle can cause TOS. Congenital and acquired etiologies such as bony anomalies (cervical ribs, transverse mega-apophyses, first rib abnormalities, and clavicle nonunions), soft tissue anomalies (cervical muscles hypertrophy or fibrous bands), and posture problems such as drooping or sagging of the shoulders or poor posture due to large breast are reported causes for TOS. 3 Common symptoms are shoulder or upper limb pain, weakness, paresthesia, and impalpable radial pulse (Raynaud's phenomenon). Surgical treatment of those lesions is often challenging due to the distorted anatomy caused by the tumor. Additionally, tumor removal and even biopsy are accompanied by risk of neurological deficits. Furthermore, total tumor resection should be performed to avoid further neurological deficits related to growth of tumor remnants. 4

This report presents our microsurgical treatment regime of brachial plexus lipomas with intraoperative electrophysiological monitoring and ultrasound assistance. Furthermore, systematic review was performed.

## Patients and Methods

Retrospective analysis of our database “peripheral nerve lesion” revealed three cases of large brachial plexus lipomas all causing neurogenic TOS (nTOS). We analyzed medical records and preoperative/postoperative magnetic resonance imaging (MRI) and performed follow-up examinations to evaluate the long-term outcome.

The study was approved by the Institutional Review Board (Medical Faculty, University of Duisburg-Essen, Registration number: 18-7955-BO).

### Systematic Review of the Literature

Systematic review was performed for literature published until March 31, 2020, analyzing PubMed, Google Scholar, Scopus, and the Cochrane Collaboration Library independently by two authors. Search keywords comprised “lipoma,” “brachial plexus, brachial plexus neuropathies or brachial plexus neuropathy.” Inclusion criteria were articles published in English presenting the clinical course, peripheral nerve location, and the treatment regime.

**Fig. 1:**
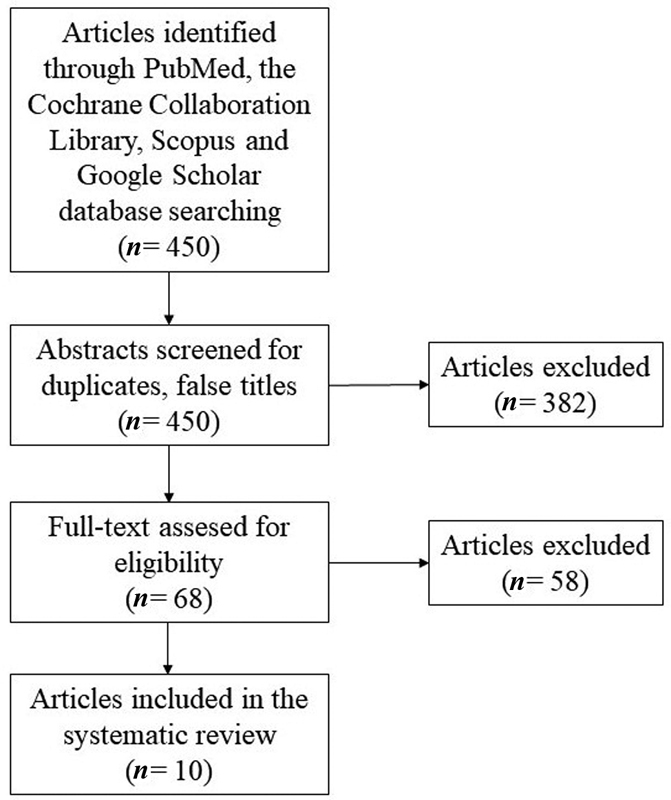
Flowchart of the systematic review.

Furthermore, the reference lists of included articles were reviewed to identify and include additional eligible articles. Those studies were meticulously cross-referenced to ensure that patients were not included in multiple articles ( Fig. 1 ). The systematic review was conducted following Preferred Reporting Items for Systematic Reviews and Meta-Analyses guidelines. 5

## Results

### Case 1

**Fig. 2:**
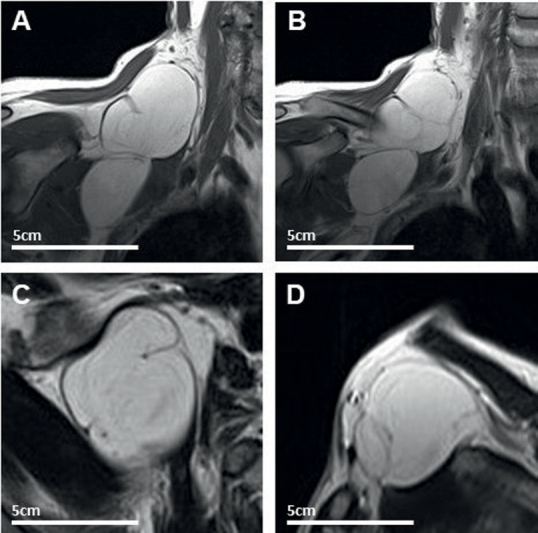
T1-weigthed imaging showing the right brachial plexus lipoma (12 × 5 cm). Coronal view ( **A** , **B** ), axial view ( **C** ), and sagittal view ( **D** ) of the lipoma.

A 62-year-old male patient presented with a pectoral subcutaneous mass lesion. The tumor showed progressive and visible growth over a period of more than 7 years, finally causing atrophy and a severe paresis of the triceps brachii muscle, the biceps brachii muscle, the deltoid muscle, and the supraspinatus muscle. Electrophysiological testing revealed an impairment of the left brachial plexus and MRI showed a large lipomatous tumor extending from the area of the triceps brachii muscle toward the supraspinatus muscle, the clavicle, and the brachial plexus on the left side (8 × 7 × 6 cm). Total piecemeal tumor resection via a two-portal supraclavicular and infraclavicular approach and neurolysis finally led to a slight improvement of the paresis on the follow-up examination 12 months after surgery ( Fig. 2 ).

### Case 2

**Fig. 3:**
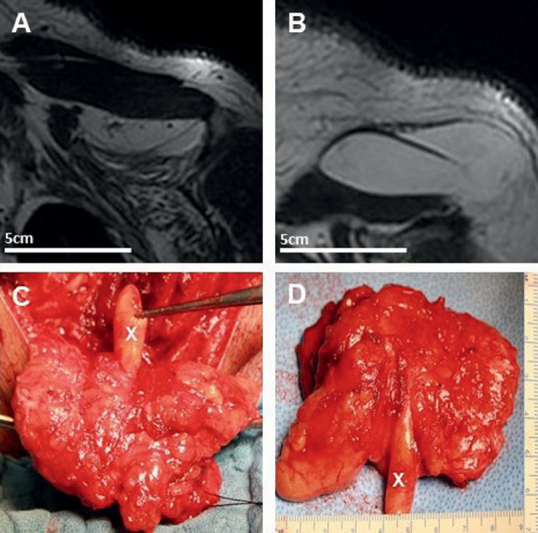
Left-sided brachial plexus lipoma (7 × 9 cm) on T2-weighted fat saturated magnetic resonance imaging ( **A** , **B** ) and intraoperative view showing a “finger-like” growth of the lipoma ( **C** , **D** ).

A 61-year-old male patient presented with a 10-year history of right-sided TOS causing hypoesthesia of digitus IV and V, a severe paresis of the triceps brachii muscle, the biceps brachii muscle, and the dorsal and palmar interossei muscles. Ultrasound and MRI of the neck depicted a large lipomatous mass (10 × 7 × 5 cm) compressing the right brachial plexus. Electrophysiological testing showed impairment of the axillary nerve. Total tumor resection via a dorsal suprascapular, infraclavicular approach and neurolysis finally led to improvement of the hypoesthesia 4 weeks after surgery. The severe paresis did not improve until the first neurological follow-up 15 months after surgery ( Fig. 3 ).

### Case 3

A 56-year-old female patient presented with a progressive swelling of the left neck and shoulder over a period of around 2 years. Preoperative MRI revealed a left-sided brachial plexus lipoma with contact to the brachial plexus. Total tumor resection finally led to long-term relief of the symptoms. Follow-up examination 9 months after surgery revealed no neurological deficit.

### Surgery

**Fig. 4:**
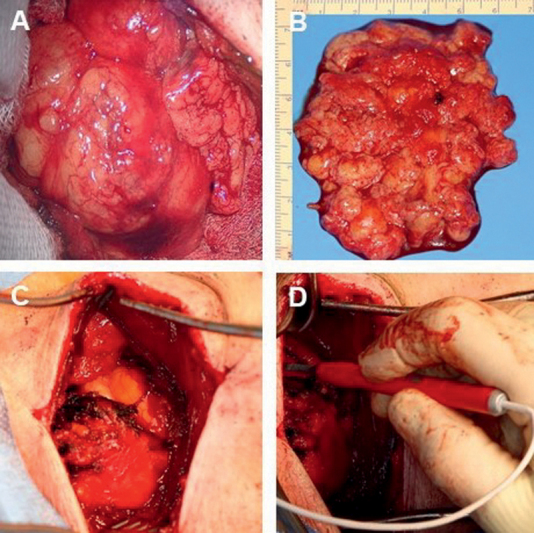
Intraoperative view on the brachial plexus lipoma (7.5 × 6.5 cm, **A** , **C** ) and after removal ( **B** ), intraoperative stimulation of the brachial plexus ( **D** ).

The patient was positioned in supine position and ultrasonography showed the supra- and infraclavicular lipomas under the pectoral muscle. Skin incision along the pectoral muscle to the axilla was followed by subcutaneous preparation toward the fascia of the muscle. Further preparation in the infraclavicular fossa visualized the lipoma beneath the clavicula. The capsule of the lipoma was then mobilized microsurgically. Further preparation was achieved via a second skin incision above the clavicula. Bipolar electrical stimulation (microfork probe 45 mm straight, Inomed, Emmendingen, Germany) was performed at the medial parts of the lipoma to test each nerve structure proximally and distally with voltages from 2 to 5 mA showing a vivid motor response. Mobilization of the tumor capsule was followed by piecemeal removal of the lipoma toward the brachial plexus using the Sonoca 300 (Söring GmBH, Quickborn, Germany) after nerve stimulation. A hemostasis procedure and subsequent removal of the lesion were followed, using bipolar coagulation. At the end, total tumor resection was achieved, and electrophysiological nerve stimulation showed that the displaced brachial plexus was not harmed ( Fig. 4 ).

### Results of the Systematic Review

Database search revealed 450 articles. Total 382 articles were excluded after analyzing the title and the abstract. The full text of the remaining 68 articles was reviewed and 58 articles were excluded afterward. In total, 10 articles were included, 2 6 7 8 9 10 11 12 13 14 comprising 22 cases of brachial plexus lipoma. Including our three cases, demographics, surgical treatment, and the neurological outcome were analyzed for 25 patients (15 males and 10 females with mean age of 46.7 ± 15.3 years). Symptom duration was 32.5 ± 39.5 months. Presenting symptoms were sensory deficit in 13 patients, an enlarged mass in 9 patients, while 8 patients complained of radiating pain. Paresis was present in five patients. Symptoms were multiple in 10 patients. In all cases, complete resection of the lipoma was possible. Neurological outcome was unchanged in one patient and improved in three patients. Five patients were asymptomatic pre- and postoperatively, and the complete relief of the symptoms was seen in 13 cases at last follow-up (mean follow-up: 15.6 ± 19.8 months). Recurrence was detected in none of the 16 reported cases. However, in three included cases, re-excision was performed due to incomplete resection in another surgical center and continuing tumor growth ( Table 1 ).

**Table 1:** Published cases of brachial plexus lipoma

Year	Reference	Age	Sex	Presentation	Symptom duration	Therapy	Postoperative outcome	Follow-up	Recurrence
2003	Sergeant et al 12	70	M	High subpectoral pain, irradiating to the left upper arm and shoulder, paresthesia, episodic dullness of all left fingers and a feeling of heaviness and minor swelling of the complete left arm	4 y	Complete resection	Complete relief	5 wk	No
2005	Vandeweyer and Scagnol 14	62	F	Enlarging mass	6 y	Complete resection	Asymptomatic	1 y	No
2007	Châtillon et al 6	44	F	Right upper extremity dysesthesias and paresthesia radiating from the shoulder to the index and middle fingers	nm	Complete resection	Complete relief	3 y	No
2009	McKay et al 11	31	M	Increasing shoulder pain and right forearm paresthesia	1 y	Complete resection	Complete relief, but mild residual right eye ptosis	1 mo	No
2011	Guha et al 9	25	M	Pain and noticeable weakness in the right arm	1 y	Complete resection	Complete relief	nm	nm
2014	Kuyumdzhiev et al 10	64	F	Numbness and tingling in the right hand and fingers with generalized weakness	3 mo	Complete resection	Complete relief	3 mo	No
		39	M	Altered sensation in the medial aspect of the right arm with pins and needles in the ulnar nerve distribution affecting right hand	nm	Complete resection	Alleviation of sensory deficit	5 mo	No
	Nakamura et al 2	47	M	Enlarging mass	4 mo	Complete resection	Asymptomatic	6 y	No
		42	F	Tenderness in the left shoulder and numbness in the lateral left upper arm	2 mo	Complete resection	Complete relief	28 mo	No
2015	Elia et al 7	30	F	Subpectoral and shoulder pain, right arm swelling, right forearm paresthesia	6 mo	Complete resection	Asymptomatic	6 mo	No
2019	Graf et al 8	58	M	Shoulder paresthesia	nm	Mass excision	Complete relief	nm	nm
		64	F	Left shoulder paresthesia and hand intrinsic weakness	nm	Excision	Complete relief	nm	nm
		49	M	Enlarging mass	nm	Excision	Asymptomatic	nm	nm
		50	M	Enlarging mass	nm	Reexcision	Asymptomatic	nm	No
		26	F	Shoulder numbness and pain	nm	Excision	Nm	nm	nm
		52	F	Shoulder paresthesia, enlarging mass	nm	Excision	Complete relief	nm	nm
		45	M	Enlarging mass	nm	Excision	Nm	nm	nm
		30	M	Painful enlarging mass	nm	Excision	Complete relief	nm	nm
		24	F	Recurrent shoulder pain, recurrent enlarging masses	nm	Reexcision	Unchanged	nm	No
		61	M	Enlarging masses	nm	Excision	Nm	nm	nm
		80	M	Recurrent pain in axilla	nm	Reexcision	Complete relief	nm	No
2019	Sul et al 13	45	M	Paresthesia and tingling sensation of the left arm	3 mo	Complete resection	Complete relief	1 y	No
	Case 1	62	M	Causing atrophy and a severe paresis of the triceps brachii muscle, the biceps brachii muscle, the deltoid muscle and the supraspinatus muscle	7 y	Complete resection	Slight improvement of the paresis	12 mo	No
	Case 2	61	M	Hypoesthesia of digitus IV and V, a severe paresis of the triceps brachii muscle, the biceps brachii muscle and the dorsal and palmar interossei muscles	10 y	Complete resection	Improvement of hypoesthesia	15 mo	No
	Case 3	56	F	Progressive swelling of the left neck and shoulder	2 y	Complete resection	Complete relief	9 mo	No

Abbreviations: MRI, magnetic resonance imaging; nm, not mentioned; nTOS, neurogenic thoracic outlet syndrome; TOS, thoracic outlet syndrome.

## Discussion

Three variants (venous, arterial, and neurological) of TOS have been described depending on the underlying pathophysiology. 4 The heterogeneity of the symptoms related to this condition makes its diagnosis challenging and treatment is still controversial. 15 The incidence is estimated to be 1 to 2% 16 with a female-to-male ratio of up to 4:1. 17 However, nTOS generally affects the brachial plexus, representing 95% of overall TOS occurrences. 18 Rinehardt et al analyzed the surgical treatment of TOS between 2005 and 2014 using the dataset of the American College of Surgeons National Surgical Quality Improvement Program. They identified 1,431 patients treated surgically (83% for nTOS, 3% for arterial TOS and 12% for venous TOS, and subtype not identifiable in 2%). Surgery was performed in 1,286 cases by a vascular surgeon, in 100 cases by a general surgeon, in 38 cases by a thoracic surgeon, in 3 cases by a neurosurgeon, in 2 cases by an orthopaedic surgeon, and in 1 case by a cardiac surgeon and a plastic surgeon. 19 Peek et al showed, in their systematic review of the literature, a 28.3-point improvement of the preoperative Disabilities of the Arm, Shoulder, and Hand score in nTOS and an overall improvement of symptoms in 56 to 89%. Complication rates ranged between 5 and 40%, with pneumothorax, nerve injuries, and wound infections being the most common complications. 20

An extremely rare cause for nTOS is large lipoma adjacent to the brachial plexus, usually presenting as a subcutaneous mass and upper limb or shoulder pain. Lipomas are benign, slow-growing soft tissue tumors and the most common mesenchymal tumor with an estimated incidence of 10%. 21 This is supported by our systematic review of the literature, showing that 40.9% of the cases were detected due to its growth as a palpable mass and 59.1% of the patients reported only sensory deficits. Symptom duration varied between 2 months and 10 years. Slow growth pattern of that benign tumor and a slow onset on neurological deficits may be the reason for late diagnosis. Furthermore, adoption of the slow deterioration of neurological deficits and therefore delayed appointment to a physician was detected in our presented cases. Additionally, delayed transfer to a neurologist performing electrophysiological diagnostic or delayed MRI may be reasonable for prolonged diagnosis.

They can arise adipose tissue in any part of the body. Lipomas are commonly located in the back, thigh, forearm, chest, and the posterior neck 22 23 and are usually solitary lesions. They present at multiple sites in 5 to 10% or rarely as a diffuse lipomatosis in a multisystem syndrome. 24 Sanchez et al defined giant lipomas as lesions more than 10 cm in size or more than 1,000 g weight. 1 The presence in various internal organs, such as liver, kidney, and lung, is also reported, although there is no or very little adipose tissue. Rarely, giant lipomas can cause pain and nerve compression syndromes. 23 The pathophysiology of tumor growth in lipomas remains unclear. Traumata seem to have an impact on the pathogenesis of lipoma. The axillary region is one of the most moveable areas of the body and microtraumata can occur with each movement of the upper limb. 25 Some authors propose that the proliferation of adipose tissue is caused by the rupture of the fibrous septa with a resulting migration of adipocytes, accompanied by tears of the anchorage between the skin and the deep fascia. 26 An alternative theory is that lipomas are resulting from preadipocyte differentiation and proliferation mediated by cytokine release followed by soft tissue damage after blunt trauma and hematoma formation. 25 27 Several histologically distinct subtypes have been described: lipoblastomas (immature vs. mature adipocytes), hibernoma (brown fat vs. white fat), angiolipoma (microvascular thrombosis present), spindle cell lipoma (mature fat cells with spindle cells and strands of dense collagen), and pleomorphic lipoma (“floret-like cells” present). 24 Differential diagnoses of giant lipomas include lipoblastomas and liposarcomas. Liposarcomas are the most common soft tissue sarcomas and account for 7 to 27% of all soft tissue sarcomas. 28 It is impossible to distinguish between lipomas and liposarcomas by the clinical appearance alone. Therefore, it is mandatory to rule out malignancy by surgical biopsy. Sarcomatous transformations of giant lipomas have been reported, but they are extremely rare. 22 Malignant transformation was not reported in the included cases of the systematic review.

Lipomas should be differentiated from other brachial plexus tumors. Most common benign brachial plexus tumors are schwannomas and neurofibromas. 29 Schwannomas are well encapsulated and characteristically form an eccentric oval swelling. The fascicles of the nerve are spread over its surface. 30 They are normally hyperintense on T2-weighted MRI with cystic formations with a strong uniform contrast enhancement. Contrary to that, neurofibromas present a circular growth pattern. The fascicles are going through the tumor. On T2-weighted MRI, neurofibromas are iso- to hyperintense with a strong partial inhomogeneous contrast enhancement. 31

Nonoperative and surgical approaches have been described as therapy of TOS. 3 Conservative treatment including physical therapy mainly focuses on strengthening and stretching of shoulder girdle elevator and pectoral muscles. 32 Even though conservative treatment may be effective in patients with posture problems, it remains ineffective in cases with structural anomalies as it can, at best, delay surgery. 3 Lipomas usually have a well-defined fibrous capsule and normally they can be resected easily by dissection around the lesions. 22 In some cases, they can be squeezed out through short incisions. 33 Liposuction of the lipoma through tiny incisions is performed by some authors even in cases of large lesions. 34 35 Possible complications of liposuction are hematomas, or in cases of incomplete resection, recurrence of the lipoma. 34 In our review, recurrence was not reported in none of the 16 reported cases, but 3 patients (12%) had to undergo re-excision after incomplete removal. Some authors favor a minimal invasive endoscopic approach via a small skin incision to remove the lipoma in total to avoid long skin incisions. 36 In our opinion, a computed tomography-guided biopsy of the lipoma should not be performed. It carries the risk to displace tumor cells after violating the capsule of the lipoma. Furthermore, complete tumor removal leads to a decompression of the brachial plexus and to a relief of the symptoms.

Lipomas adjacent to major nerves and vessels should be removed by microsurgical excision with electrophysiological neuromonitoring to prevent neurological deficits. Blind aspiration may injure these structures, resulting in a major bleeding or neuromuscular dysfunction with paralysis.

Although advances in surgical strategies including peripheral nerve microsurgery have improved the outcome, there is still a risk of causing intraoperative damage. Preserving the neurological function while trying to achieve gross total resection should be the principle treatment goal. Therefore, intraoperative neurophysiological monitoring combined with intraoperative high-resolution ultrasound represents an effective technique for identifying and monitoring functional integrity of both the spinal cord and the nerve roots in real time. The benefit of intraoperative neurophysiological monitoring for preserving neuronal structures and achieving an optimal postoperative functional outcome was proven in several studies. 37 38 Furthermore, electrophysiological monitoring is a useful tool to test nerve integrity and to determine the topography of the injury site. 39

In our opinion, microsurgical resection with intraoperative electrophysiological monitoring should be performed to remove the tumor in total and to avoid nerve injuries.

## Conclusion

Brachial plexus lipomas are an extremely rare cause for TOS and are typically diagnosed several months delay after the initial symptoms. Microsurgical removal under permanent intraoperative electrophysiological monitoring should be the treatment of choice as it is safe and has a favorable outcome.
